# Flow of affective information between communicating brains

**DOI:** 10.1016/j.neuroimage.2010.07.004

**Published:** 2011-01-01

**Authors:** Silke Anders, Jakob Heinzle, Nikolaus Weiskopf, Thomas Ethofer, John-Dylan Haynes

**Affiliations:** a Department of Neurology and Neuroimage Nord, University of Lübeck, Lübeck, Germany; b Bernstein Center for Computational Neuroscience Berlin, Charité-Universitätsmedizin, Berlin, Germany; c Wellcome Trust Centre for Neuroimaging, UCL Institute of Neurology, University College London, London, UK; d Department of Psychiatry, University of Tübingen, Tübingen, Germany; e Max-Planck-Institute for Human Cognitive and Brain Sciences, Leipzig, Germany

**Keywords:** fMRI, Decoding, Emotion, Communication, Facial expression, Embodied simulation

## Abstract

When people interact, affective information is transmitted between their brains. Modern imaging techniques permit to investigate the dynamics of this brain-to-brain transfer of information. Here, we used information-based functional magnetic resonance imaging (fMRI) to investigate the flow of affective information between the brains of senders and perceivers engaged in ongoing facial communication of affect. We found that the level of neural activity within a distributed network of the perceiver's brain can be successfully predicted from the neural activity in the same network in the sender's brain, depending on the affect that is currently being communicated. Furthermore, there was a temporal succession in the flow of affective information from the sender's brain to the perceiver's brain, with information in the perceiver's brain being significantly delayed relative to information in the sender's brain. This delay decreased over time, possibly reflecting some ‘tuning in’ of the perceiver with the sender. Our data support current theories of intersubjectivity by providing direct evidence that during ongoing facial communication a ‘shared space’ of affect is successively built up between senders and perceivers of affective facial signals.

## Research highlights

►Information-based neuroimaging is used to map flow of affective information between brains. ►Information is encoded in a ‘shared network’ in the brains of senders and perceivers. ►Dynamics of information flow suggest some ‘tuning-in’ of the perceiver with the sender.

## Introduction

Exchange of information between brains is essential for successful human interaction. Interaction partners must continuously update information about their partner's inner state, intentions, motivation and affect, in order to anticipate the other one's behaviour and to adapt their own behaviour accordingly. One mechanism that has been proposed to play an important role in exchange of affective information between individuals is ‘embodied simulation’ (Gallese, 2003; see also Lipps, 1903; Adolphs et al., 2000; Decety and Jackson, 2004; Bastiaansen et al., 2009; Iacoboni, 2009). The idea is that when people observe another person's affective behaviour, their facial expression, gesture or movement, this automatically activates a ‘mirror’ representation of the other person's affect in the perceiver's brain. In other words, experiencing and perceiving affect are thought to activate similar neural networks, creating a ‘shared space of affect’ between senders and perceivers of affective information.

A number of neuroimaging studies have found evidence that is consistent with the idea that ‘embodied stimulation’ plays a role in the exchange of affective information between brains. For example, it has been shown that when volunteers observe another person receiving a painful stimulus they activate part of a ‘pain network’ that is also activated when the volunteers themselves receive a painful stimulus (Morrison et al., 2004; Singer et al., 2004; Botvinick et al., 2005). Other studies provide evidence that a region in the anterior insula is recruited both when people experience disgust and when they observe another person experiencing disgust (Calder et al., 2000; Wicker et al., 2003). More generally, it has been suggested that observing a facial expression of affect activates part of a somato-motor network that is also activated when volunteers express their own affect (Carr et al., 2003; Leslie et al., 2004; Hennenlotter et al., 2005; van der Gaag et al., 2007).

However, it has to date remained unclear whether these networks indeed carry similar affect-specific information in the sender and perceiver. Moreover, if ‘embodied simulation’ plays a role in the brain-to-brain transfer of affective information, then there should be a temporal succession of information in these networks from the sender to the perceiver. These temporal dynamics can only be studied by comparing brain processes in two individuals engaged in ongoing affective communication. We used information-based functional magnetic resonance imaging (fMRI) (Haxby et al., 2001; Haynes and Rees, 2006; Kriegeskorte et al., 2006; Norman et al., 2006) to directly investigate the dynamics of the flow of information between the brains of senders and perceivers engaged in facial communication of affect. First, we aimed to identify a ‘shared network’ of affect that carries similar *information* in both the sender and the perceiver that is *specific to the emotion* that is currently being communicated. Second, we tested whether there was a *temporal succession* in the flow of information from the sender's brain to the perceiver's brain.

## Methods

### Participants

Because it has been suggested that exchange of affective information is strongest between closely attached individuals (Singer et al., 2004), we investigated the flow of affective information between romantic partners. Six different-sex couples (mean age of women 22 years, range 20 to 25 years, mean age of men 24 years, range 22 to 28 years) who had been engaged in a romantic relation for at least one year at the time of scanning (mean 2 years, range 1 to 4 years) participated in the study. All participants were right-handed and reported no history of neurological or psychiatric disorders. Participants gave their written informed consent prior to participation and the study was approved by the local ethics committee.

### Experimental design

Partners were invited together to the scanning facility. After a brief introduction, they were informed that they would be scanned simultaneously and that the perceiver would see the sender's facial expression online during scanning via a video-camera. Partners were then separated and the female partner was informed that her task would be to indulge herself into emotional situations and to facially express her emotional feelings as soon as they arose. The female partner was selected as sender because women have been shown to be more accurate senders of affect than men (Buck et al., 1974). Particular care was taken to ensure that the sender understood that she was not meant to pose emotional expressions but to try to share her emotional feelings with her romantic partner as they arose. The male partner was completely uninformed about the sender's task and was simply asked to watch the senders' facial expression and to try to feel with her (i.e. the male partner did not know that the sender was asked to submerge herself into emotional situations, please see Supplemental online material for the wording of the instructions). In fact, the sender's facial expression was videotaped throughout scanning and shown to the perceiver when he was scanned in the same scanner immediately after scanning of the sender had been completed.

**Fig. 1:**
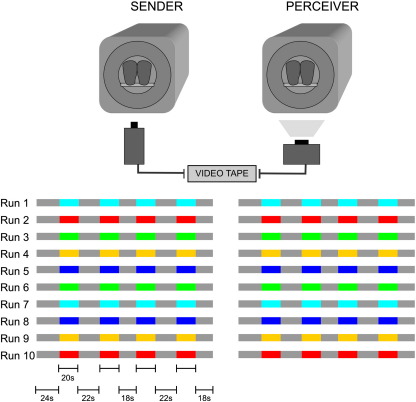
Experimental design. Senders and perceivers participated in 10 runs of fMRI. Colours indicate emotion periods; each colour indicates a different emotion (joy, anger, disgust, fear, or sadness), grey indicates resting periods. The order of runs was chosen by the sender with the restriction that each emotion had to be chosen once before an emotion could occur a second time. The sender's facial expression was video-taped throughout scanning and shown to the perceiver while he was scanned immediately after scanning of the sender had been completed.

Scanning consisted of ten runs; each run comprised four 20 s-periods during which affective information was to be communicated, and five interspersed periods during which the sender was instructed to relax (24 s, 22 s, 18 s, 22 s, and 18 s) (Fig. 1). A single emotion (joy, anger, disgust, fear, or sadness) was used in each run in order to avoid rapid switches between conflicting emotions. A single printed word (e.g. ‘joy’) signalled emotion periods to the sender. The order of emotions was chosen by the sender, with the restriction that no emotion could occur twice in a row and that each emotion had to be chosen once before an emotion could be chosen a second time. Please note that the perceiver was uninformed about the timing within runs.

### Data acquisition

Ninety-two (92) functional images covering the whole brain were acquired during each run (T2*weighted echoplanar images, 1.5 Tesla Siemens Avanto, Erlangen, Germany; tilt angle −30°, 64 × 64 matrix, in plane resolution 3 × 3 mm², 24 axial slices, interleaved order, slice thickness 6 mm with no gap, TE 40 ms, TR 2000 ms). An fMRI-compatible video camera (Wild et al., 2000) was used to video-tape the sender's facial expression throughout scanning. Additionally, we recorded skin conductance responses (SCR) as a peripheral index of autonomic activity during scanning with standard commercial recording equipment (Varioport, Becker Meditec, Karlsruhe, Germany). Details of skin conductance data acquisition have been described elsewhere (Anders et al., 2004). Stimulus presentation and data collection were synchronised with Presentation software (Neurobehavioral Systems Inc., Albany, CA, USA). After each run, perceivers were asked via the intercom what they thought the sender might have been feeling, and if they thought that they had felt the same as the sender, and responses were protocolled word-by-word by one of the experimenters. Skin conductance responses and verbal reports served to confirm that participants showed emotional engagement during emotion periods. No explicit emotion recognition data were collected in order to avoid subject priming.

### Analysis of behavioural data

Usable SCR data were obtained from three senders and five perceivers. Data of the remaining individuals could not be analysed due to recording errors. For analysis, linear trends were removed from the time series of each run, and the average level of SCR during the 20 s-emotion periods was contrasted the average of a 4 s rest period before the onset of each emotion period.

Word-by-word protocols recorded after each run were used to derive a measure of emotion recognition accuracy for each run. Emotion recognition was parameterized as '1' if the perceiver correctly named the sender's emotion, or gave a description that was correctly identified by two independent raters, and '0' otherwise. Chi-Square statistics tested for differences in emotion recognition between emotion types.

### Analysis of fMRI data

Preprocessing of functional images included slice acquisition time correction, concurrent spatial realignment and correction of image distortions by use of individual static field maps (Andersson et al., 2001), normalization into standard MNI space (Montreal Neurological Institute) and spatial smoothing (10 mm Gaussian kernel) (SPM5, Wellcome Department of Imaging Neuroscience, London, UK).

#### Voxel-wise classification analysis

Previous studies have shown that levels of activity within a distributed network of brain regions differ, depending on the individual's affective state (e.g. Damasio et al., 2000; Phan et al., 2002). Thus, to identify a ‘shared network’ for affective information we searched for voxels where the *level of emotion-specific activity* in the sender's brain was reflected in the perceiver's brain. For this purpose, we first computed an image of voxel-wise parameter estimates for each emotion period (i.e. each trial) using a general linear model as implemented in SPM5. This resulted in 40 parameter estimates (4 trials per run × 10 runs) for each voxel and participant. To prewhite data for the classification analysis, the global mean was subtracted from each image and the 40 parameter estimates for a given voxel were normalized to zero mean and unity across all emotion periods.

Classification analysis was carried out separately for each voxel. First, in a given voxel, mean values were computed across the eight parameter estimates of each emotion (4 trials per run × 2 runs per emotion) for the sender (‘Training’). Second, these mean values were used to classify the perceiver's brain activity. The perceiver's brain response in a given emotion period was classified according to the smallest distances between the perceiver's brain response and these mean values (‘Test’) (Fig. S1 Supplemental online material). This approach is similar to a univariate k-nearest-neighbour classification except that classification is based on the nearest mean value instead of k nearest neighbours. This yielded a total of 40 binary classification accuracies per voxel. To derive a single measure of decoding accuracy per voxel for each sender-perceiver pair, voxel-wise classification accuracies were averaged across all trials.

For group statistical inference, the images of voxel-wise decoding accuracies were subtracted with chance level (p = .20), spatially smoothed (10 mm Gaussian kernel) to allow for Random Field Theory statistical inference (Worsley et al., 1996), and fed into a one-sample T-test with random factor subject as implemented in SPM5. The resulting statistical parametric map (SPM) tested the H_0_ that decoding accuracies were not greater than chance. Because previous work has shown that single voxels carry limited information only and that information is be encoded in extended brain regions (Pessoa and Padmala, 2007) we used a voxel-wise height threshold of T = 3.3 (corresponding to a probability of false positives of p = .01) and assessed statistical significance at cluster level (p = .01, corrected for multiple comparisons according to Random Field Theory [Worsley et al., 1996]; this corresponded to a minimal cluster size of 100 contiguous voxels). Please note, however, that the number and location of clusters was highly stable across different height thresholds.

#### Time-resolved classification analysis

Next, we sought to investigate the temporal dynamics of the flow of affective information from the sender's brain to the perceiver's brain. Particularly, we were interested whether early information from the sender's brain was encoded early in the perceiver's brain, and late information from the sender's brain was encoded later in the perceiver's brain. This would indicate that there was a temporal succession in the flow of emotion-specific information from the sender's brain to the perceiver's brain. For this purpose we used a time-resolved multivariate decoder. As before, a classifier was trained on the sender's brain activity and tested on the perceiver's brain activity. However, the time-resolved classification analysis was based on intensity values in single functional images. After preprocessing, global means were removed from each image and voxel-wise intensities were normalized as described above. Functional images were then temporally aligned with respect to the onset of an emotion period. This resulted in 40 time series (10 runs × 4 emotion periods per run) of 18 functional images per subject, each covering a 36 s-time interval from 2 scans before the onset of an emotion period to 6 scans after offset of the emotion period.

Because we were interested in the temporal dynamics with which information in voxels known to carry emotion-specific information (i.e. the ‘shared network’) was transferred from the sender to the perceiver we aimed to restrict the analysis to these voxels. To avoid circularity, we identified voxels that carried emotion-specific information separately for each sender–perceiver pair, based on data from the remaining sender–perceiver pairs only. Thus, for each sender–perceiver pair the most significant 2500 voxels in the group SPM derived from the remaining sender–perceiver pairs were selected (corresponding roughly to the number of above-threshold voxels in the voxel-wise analysis).

For classification, the intensity values of these voxels within a 2 s-time window (corresponding to one functional scan) were represented as a vector in m-dimensional space, where m is the number of voxels. Classification was based on Euclidian distances between vectors. First, for each 2 s-time window, mean vectors were computed across the eight intensity vectors of each emotion (4 trials per run × 2 runs per emotion) for the sender (‘Training’). Second, these mean vectors were used to classify the perceiver's brain activity. The perceiver's response in a given 2s-time window was classified according to the smallest Euclidian distances in m-dimensional space between the perceiver's response and these mean vectors (‘Test’) (Fig. S2 Supplemental online material). Classification of the perceiver's brain activity was carried out separately for each time window of the sender's brain activity and each time window of the perceiver's brain activity, and separately for each emotion period. This yielded a total of 40 binary classification accuracies for each combination of time windows. To derive one measure of decoding accuracy for each combination of time windows, classification accuracies were averaged across all emotion periods. This resulted in an n-by-n matrix of time-resolved decoding accuracies for each sender–perceiver pair, where n is the number of time windows. Each row of the time-resolved matrix of decoding accuracies represents the time course f_i_(p_i_) with which information from the sender's brain in a specific 2 s-time window s_i_ was encoded in the perceiver's brain.

To extract the dynamics of information flow from the time-resolved matrices, time-resolved matrices were first temporally smoothed with a filter width corresponding the time course of the hemodynamic response function (4s × 4s Gaussian filter). Then, time courses were averaged across all time windows s_i_ and this average was subtracted from each individual time course. This resulted in a new n-by-n matrix for each sender–perceiver pair. For each time course f′_i_(p_i_) in this new matrix we determined the time window p_imax_ in which information from the sender's brain in time window s_i_ was most accurately encoded in the perceiver's brain [i.e. f′_i_(p_imax_) = max(f′_i_(p_i_))]. To test whether there was a temporal succession in the flow of information (i.e. whether there was a positive linear relation between time windows s_i_ and p_imax_) we used a linear contrast within a repeated-measures ANOVA with fixed factor time window of the senders brain activity and random factor subject. Because we were mainly interested in the flow of *affective* information, the ANOVA was restricted to the time interval covered by the expected time course of the hemodynamic response during the affective communication period (i.e. from 2 scans after beginning of an emotion period to 2 scans after the end of an emotion period). The delay between information in the sender's brain and information in the perceiver's brain for a given time window s_i_ was defined as p_imax_ − s_i_. A linear contrast within a repeated-measures ANOVA with fixed factor time window of the senders brain activity and random factor subject was used to test for changes of delay over time. Average group matrices and time courses are shown for visualisation (Fig. 3), but analyses of the time course of information flow were carried out separately for each sender–perceiver pair and statistical analyses are based on data of individual sender–perceiver pairs.

#### Supplemental analysis

As stated above, all of the above analyses were carried out within true sender–perceiver pairs. To test the possibility that the ‘shared network’ carried information in individual sender–perceiver pairs that was specific to each sender–perceiver pair and that exceeded information that was present in all senders and perceivers, we performed an additional analysis that compared classification accuracies within true sender–perceiver pairs to classification accuracies within arbitrary sender–perceiver dyads. To this end we combined information across all m voxels in the shared network as described above. However, because this time we were not interested in the time course with which information was transferred, vectors in m-dimensional space now represented m parameter estimates as in the first analysis (rather than m intensity values from single functional images). Because we reasoned that information that was specific to specific sender–perceiver pairs would vary across trials we performed a separate classification analysis for each trial. First, a vector was computed for each trial for the sender (‘Training’). The perceiver's response in a given emotion period was then classified according to the smallest Euclidian distance in m-dimensional space between the perceiver's response and the five vectors representing the sender's emotion-specific response during the corresponding trials (‘Test’). This resulted in 40 classification accuracies for each sender–perceiver pair. We then repeated the same analysis, this time pairing each sender with each perceiver except her true communication partner, and each perceiver with each sender except his true communication partner. To derive one sender – other-perceivers classification accuracy for each sender, and one other-senders – perceiver classification accuracy for each perceiver, classification accuracies were averaged across perceivers and senders, respectively. To avoid circularity, voxels that carried emotion-specific information were identified separately for each sender–perceiver pair, based on data from all remaining sender–perceiver pairs only.

## Results

### Skin conductance and emotion recognition

Average time series of skin conductance responses of senders and perceivers are shown in Fig. S3 Supplemental online material. In both senders and perceivers SCR increased during emotion periods (senders, .63 ± .07 μS [mean ± s.e.m.]; perceivers, .09 ± .02 μS; all participants, T(7) = 2.6, p < .05; perceivers only, T(4) = 3.9, p < .01). This indicates that affective communication led to an increase of autonomic arousal. Furthermore, in line with the assumption that the perceiver's autonomic response reflected the sender's autonomic response over time, visual inspection of the time series indicates that the increase in perceivers was delayed relative to the increase in senders.

Perceivers recognized the sender's emotion in 70 percent of the runs. Recognition rates were highest for joy (1.00) and lowest for anger (.50), but recognition was above chance (.20) for each and every type of emotion (binomial p <= .05 for all types of emotion), and there was no statistically significant difference in recognition rates between emotion types (Chi-Square = 2.5, df = 4, p > .50, Fig. S4 Supplemental online material).

### A shared network of affect

**Fig. 2:**
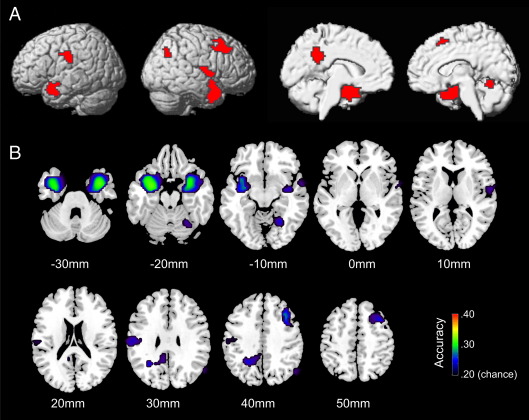
The ‘shared network of affect’. A. Clusters in which the perceiver’s brain activity could successfully be predicted from the level of the sender’s brain activity, depending on the communicated affect (p = .01, corrected for multiple comparisons at cluster level). Significant clusters are projected onto the surface of a standard brain (MNI). B. Average voxel-wise decoding accuracies within each cluster, projected onto axial slices of the same brain as in A. Slices are shown in neurological convention (left is left). Numbers below slices indicate z coordinates.

First, we aimed to identify brain regions where the perceiver's brain activity could be predicted from the sender's brain activity, depending on the specific emotion that was currently being communicated. For this purpose we trained a simple univariate classifier to identify the sender's current emotion based on the level of activity in a given voxel in the sender's brain, and tested whether the same classifier could identify this emotion from the level of activity in the same voxel in the perceiver's brain. This procedure revealed that the perceiver's emotion-specific brain activity could successfully be predicted from the sender's emotion-specific brain activity in a distributed network, including temporal, parietal, insular and frontal brain regions (Fig. 2 and Table S1 Supplemental online material). In other words, these brain regions carried highly similar information in the sender's and perceiver's brain, and this information was encoded by highly similar signals in the sender's and perceiver's brain.

### Dynamics of information flow

Next, we sought to investigate the temporal dynamics of the flow of affective information. Particularly, we were interested whether early information from the sender's brain was encoded early in the perceiver's brain, and late information from the sender's brain was encoded later in the perceiver's brain. This would indicate that there was a temporal succession in the flow of emotion-specific information from the sender's brain to the perceiver's brain. For this purpose we used a time-resolved multivariate decoder. As before, a classifier was trained on the sender's brain activity and tested on the perceiver's brain activity. However, this time the classifier was trained on the sender's brain activity in all voxels of the shared network within a specific 2 s-time window and then tested on the perceiver's brain activity in another 2 s-time window.

**Fig. 3:**
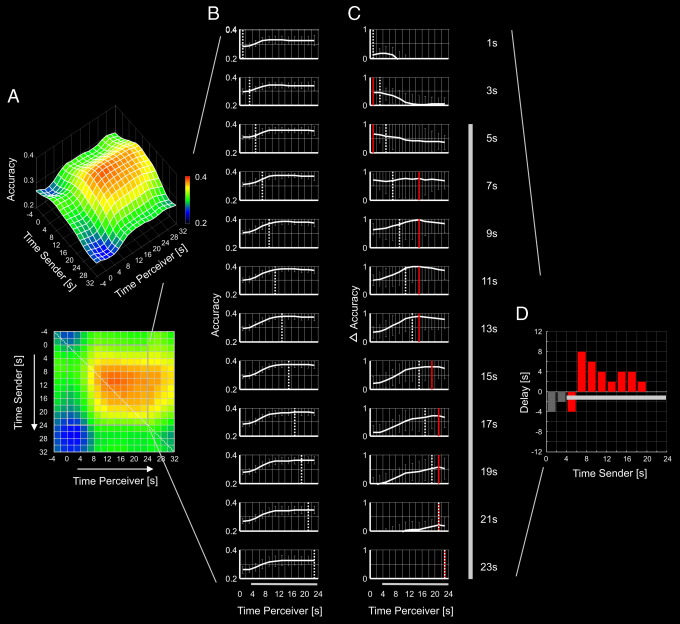
Dynamics of information flow. Each row of the time-resolved matrix of decoding accuracies (A) represents the time course (B) with which information from the sender's brain in a specific time window was encoded in the perceiver's brain. Subtracting the average from these time courses reveals the temporal dynamics of information flow (C). Time courses of delta accuracy in C are scaled to the overall maximum of delta accuracy. Red lines indicate the peak of each individual time course; dashed lines and numbers on the right indicate the time window the sender's brain activity was taken from. The bar chart (D) shows the delay with which information from the sender's brain was reflected in the perceiver's brain. Dark grey bars in C and D represent an approximation of the interval covered by the predicted time course of the hemodynamic response during affective communication.

Each row of the time-resolved matrix of decoding accuracies (Fig. 3A) represents the time course with which information from the sender's brain in a specific 2 s-time window was encoded in the perceiver's brain. Initially, these time courses all appear highly similar (Fig. 3B). This would suggest that brain activity within the shared network was specific to each emotion but did not change much over a given emotion period. Importantly, however, the temporal dynamics of information flow became visible when the average time course (i.e. the stationary component) was subtracted from each individual time course. This revealed that the accuracy with which information in the sender's brain in a specific time window was reflected in the perceiver's brain was not stationary, but changed in a systematic manner over time. Early information from the sender's brain was most accurately encoded in the perceiver's brain at early time points; and late information from the sender's brain was most accurately encoded in the perceiver's brain at later time points (T = 2.2, p < .05, one-tailed, Fig. 3C).

Fig. 3D also shows that there was a considerable delay between information in the sender's brain and information in the perceiver's brain. The bar chart shows, for each time window, the delay with which information from the sender's brain was reflected in the perceiver's brain. This delay was large (up to 8 s) just after the beginning of an affective period and decreased towards the end of an affective period (close to 0 s) (T = −3.6, p < .05, two-tailed). Please note that this delay cannot be explained by the latency of the hemodynamic response because time courses of information flow were computed from fMRI signals of the sender and the perceiver and the hemodynamic delay is thus a common component to both signals.

### Specificity of information within the shared network

**Fig. 4:**
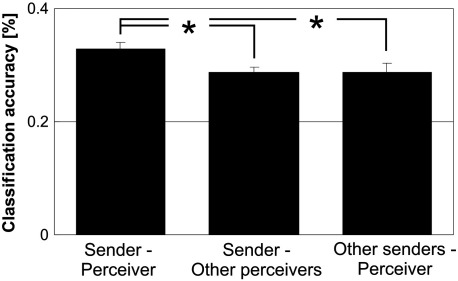
Specificity of information within the ‘shared network of affect’. Bar charts represent average classification accuracies when information was combined across all voxels within the ‘shared network of affect’. Classification accuracy was significantly lower within sender – other-perceiver dyads and other-sender – perceiver dyads than within true sender–perceiver pairs. Error bars represent standard errors of the mean.

As additional analysis, we tested whether the ‘shared network’ carried information in individual sender–perceiver pairs that was specific to each sender–perceiver pair and that exceeded information that was present in all senders and perceivers (Fig. 4). Specifically, we tested whether classification accuracies within true sender–perceiver pairs were higher than classification accuracies within sender – other-perceiver dyads and within other-sender – perceiver dyads. This was indeed the case. Classification accuracies were still above chance for sender – other-perceiver dyads (mean accuracy = .29, T = 10.1, df = 5, p < .001) and other-sender – perceiver dyads (mean accuracy = .29, T = 5.9, df = 5, p < .001), indicating that similar information was present in all senders and perceivers. Critically, however, classification accuracy was significantly lower within sender – other-perceiver dyads than within true sender – perceiver pairs (paired T-test, T = 2.3, p = .03) and significantly lower within other-sender – perceiver dyads than within true sender – perceiver pairs (paired T-test, T = 2.4, p = .03). This provides clear evidence that the shared network does not only represent ‘prototypical’ emotional information but indeed carries information about a communication partner's *individual* affective state.

## Discussion

In sum, our data show that during ongoing facial communication of affect, emotion-specific information is encoded in similar distributed networks in the sender's and perceiver's brain. Furthermore, there is a temporal succession in the flow of affective information from the sender's to the perceiver's brain, with information in the perceiver's brain being delayed relative to information in the sender's brain. These findings extend existing knowledge on the neural basis of affective communication in two important ways.

First, we show that distributed anterior temporal, insular and somato-motor brain regions that have been associated with ‘embodied simulation’ during affective communication not only show common activity during emotion observation and first-hand emotional experience (Wicker et al., 2003; Carr et al., 2003; Leslie et al., 2004; Hennenlotter et al., 2005; van der Gaag et al., 2007), but indeed carry emotion-specific information in the sender and perceiver. Moreover, our data show that this information is encoded by highly similar signals in the sender and perceiver. This can be seen from the fact that information about the specific emotion that was communicated by the sender could be decoded from the level of activity in individual voxels within this network in the perceiver's brain even though the decoder was trained on the sender's brain activity only. Thus, our analyses provide evidence for a ‘common coding’ of emotion-specific information in a distributed network in the sender's and perceiver's brain. Interestingly, the ventral premotor cortex, which is often activated when people imitate or observe posed affective facial expressions (Carr et al., 2003; Leslie et al., 2004; Hennenlotter et al., 2005; van der Gaag et al., 2007), was not part of this network.

Second, our approach allowed us to directly measure how information from the sender's brain is subsequently encoded in the perceiver's brain. Thus, we could show that there was a temporal succession in the flow of affective information, with early information from the sender's brain being encoded early in the perceiver's brain, and later information from the sender's brain being encoded later in the perceiver's brain. This illustrates that information from the sender's brain was dynamically reflected in the perceiver's brain.

A very recent study by Schippers et al. (2010) used a design similar to that in the current study except that senders in that study did not communicate their affective state, but gestured arbitrary words to the perceiver. Using between-subject Granger causality analysis that study showed that changes of activity in certain regions of the sender's brain preceded changes of activity in other regions of the perceiver's brain. The current study supports and extends those findings by showing that information about the *specific content* of communication (in this case the sender's affective state) from the sender's brain is subsequently reflected in the perceiver's brain.

Notably, there was a considerable delay between information in the sender's brain and information in the perceiver's brain. This delay (up to eight seconds) was much longer than it would be expected due to the very brief physical delay of the video signal or neuronal transmission delays in low-level motor and sensory systems in the sender and perceiver. Also, delays were computed directly from the sender's and perceiver's fMRI signal and thus cannot be explained the latency of the hemodynamic response. At first glance this result seems to be at odds with behavioural studies that have shown that covert mimicking reactions to facial and bodily expressions of emotion can occur very rapidly (e.g. Dimberg and Thunberg, 1998; van Heijnsbergen et al., 2007). However, the finding that it may take several seconds until the sender's affective state is fully reflected in the perceiver's brain is in line with the view the human emotions comprise different response components (e.g. Anders et al., 2009) that unfold over time (Leventhal and Scherer, 1987). Interestingly, the delay between information in the sender's brain and information in the perceiver's brain decreased over an affective period. This possibly reflects some ‘tuning in’ of the perceiver with the sender. In other words, while it may initially take some time for the ‘shared space’ (Gallese, 2003) of affect to build up between the sender and perceiver, information transfer seems to become faster once the shared space has been established.

Finally, our data provide evidence that the ‘shared network’ does not only carry ‘prototypical’ emotional information but indeed carries information about an interaction partner's *individual* affective state. This can be derived from the finding that classification accuracies within the shared network were higher within true sender–perceiver pairs than within arbitrary sender–perceiver dyads. This underlines a possible role of the ‘shared network’ in simulating another person's current affective state rather than in solely reflecting prototypical information.

The current study, like most previous studies, investigated ‘embodied simulation’ during facial communication in a context in which perceivers were biased to share the sender's affective state. In real-life situations this might not always be the case. In certain contexts, situational demands might lead to an inhibition of ‘embodied simulation’ (e.g. Lamm et al., 2007). The current study provides a tool to directly study such influences by assessing the amount of specific information that is reflected in the perceiver's brain, depending on the context.

One important question that has to be considered when investigating a possible role of ‘embodied stimulation’ in social interaction is how such a mechanism might be implemented at the neuronal level. A seminal finding in this regard was the detection of neurons in the macaque area F5 (a premotor area) that fire not only when the monkey performs a goal-directed hand movement, but also when the monkey observes the same action being performed by another individual (Gallese et al., 1996; Rizzolatti et al., 1996). This and subsequent findings have been taken as evidence that ‘mirror neurons’ (i.e. neurons that fire during observation and action) might link third-person observation and first-hand experience and thereby provide an important basis for insightful social interaction (e.g. Gallese, 2003). The search for such ‘mirror neurons’ in the human brain has been restricted by the limited spatial resolution of recording methods available for human research. Nevertheless, along with a single record of a ‘mirror neuron’ for pain in the human brain (Hutchison et al., 1999) there are a number of studies that have shown that some regions in the human brain respond during observation and execution of specific actions (Iacoboni et al., 1999; Shmuelof and Zohary, 2006; Dinstein et al., 2007; Gazzola and Keysers, 2009; Schippers et al., 2009), or observation and first-hand experience of affect (Wicker et al., 2003; Carr et al., 2003; Leslie et al., 2004; Hennenlotter et al., 2005; van der Gaag et al., 2007). However, there is an ongoing debate whether common activity in neuroimaging studies indeed reflects the existence of ‘mirror neurons’ in these regions. Alternatively, observed and executed actions, or observed and experienced affect, could be represented by different subpopulations of neurons within the same region (Morrison and Downing, 2007; Dinstein et al., 2008; Lingnau et al., 2009). The current study shows that affective information is represented in similar neural networks in the sender's and perceiver's brain, and that affective information within these networks is encoded by highly similar signals. It will remain a challenging task for future studies to examine the neurophysiological bases of this information transfer below the current resolution of neuroimaging studies.

In conclusion, our data support current theories of intersubjectivity by showing that affect-specific information is encoded in a very similar way in the brains of senders and perceivers engaged in facial communication of affect. Information is successively transferred from the sender's brain to the perceiver's brain, eventually leading to what has been called a ‘shared space’ of affect (Gallese, 2003). At the same time, our approach extends the individual-focussed approach of previous neuroimaging studies on the social cognition to a dyadic, inter-individual perspective. Previous studies have examined how common stimulation synchronizes brain activity of individuals (Hasson et al., 2004), and have investigated brain responses of individuals involved in ongoing social interaction (King-Casas et al., 2005; Tognoli et al., 2007), but have not directly studied how activity in one individual's brain influences, or depends on, activity in another individual's brain. However, to understand the neurobiological bases of social interaction it is fundamental to understand how the brains of individuals interact. The approach presented here, using information-based imaging to directly compare brain activity of individuals engaged in ongoing social interaction, provides a tool that might open a new perspective in social neuroscience that seeks to examine the neurobiology of human social behaviour from an inter-individual point of view.

## Author contributions

S.A., T.E. and N.W. conceived the experiment, S.A. and T.E. carried out the experiment, S.A. and J.-D.H. analysed the data, J.H. provided analysis tools, S.A. and J.-D.H. wrote the manuscript, S.A., J.H., N.W., T.E. and J.-D.H. edited the manuscript.
