# Supplementary material for: Flow of affective information between communicating brains
Source: Neuroimage. 2011 Jan 1;54(1-4):439–46. doi: 10.1016/j.neuroimage.2010.07.004 (PMC3081064; doi:10.1016/j.neuroimage.2010.07.004)
Supplement: Supplementary file 1 — Supplementary materials. [file mmc1.doc]

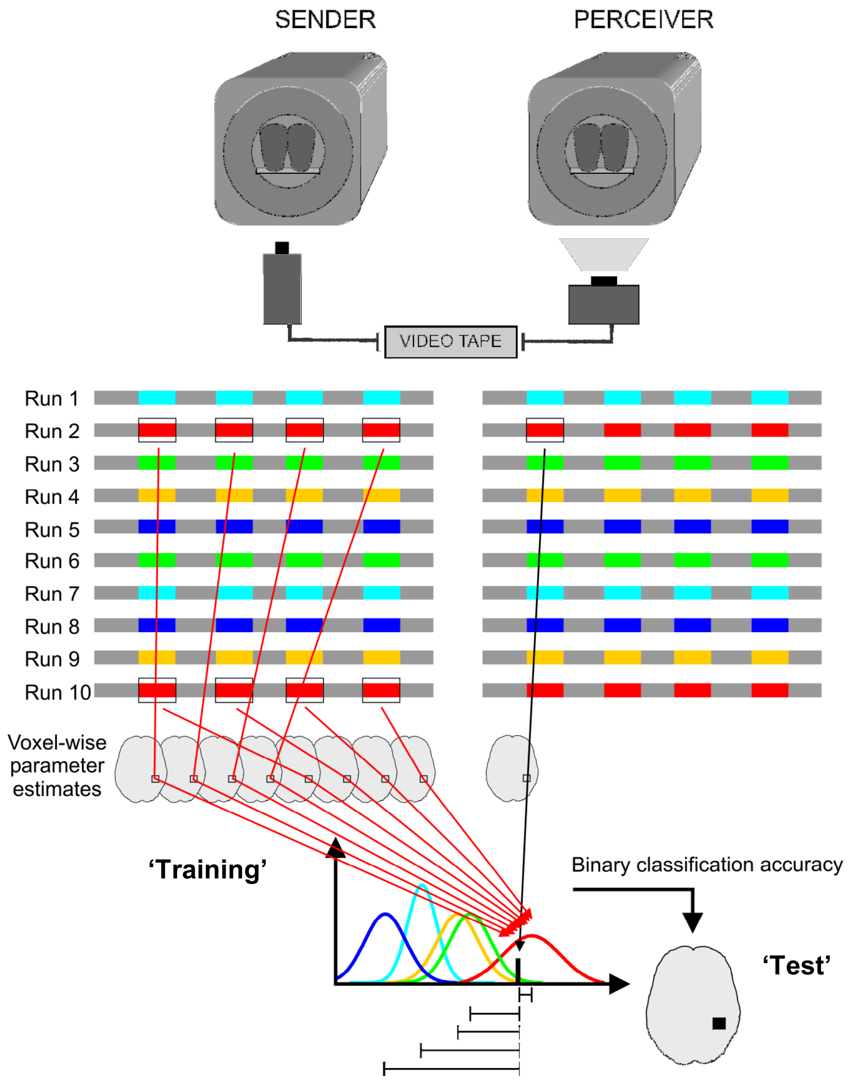

Figure S1. Voxel-wise classification analysis. Classification was based on voxel-wise parameter estimates. First, in a given voxel, mean values were computed across the eight parameter estimates of each emotion (4 trials per run x 2 runs per emotion, indicated by the same colour) for the sender ('Training'). Second, these mean values were used to classify the perceiver's brain activity. The perceiver's brain response in a given emotion period was classified according to the smallest distances between the perceiver's brain response (indicated by the black bar in the frequency distribution) and these mean values ('Test'). Classification of the perceiver's brain activity was carried out separately for each emotion period, yielding a total of 40 binary classification accuracies (10 runs x 4 emotion periods per run) per voxel.

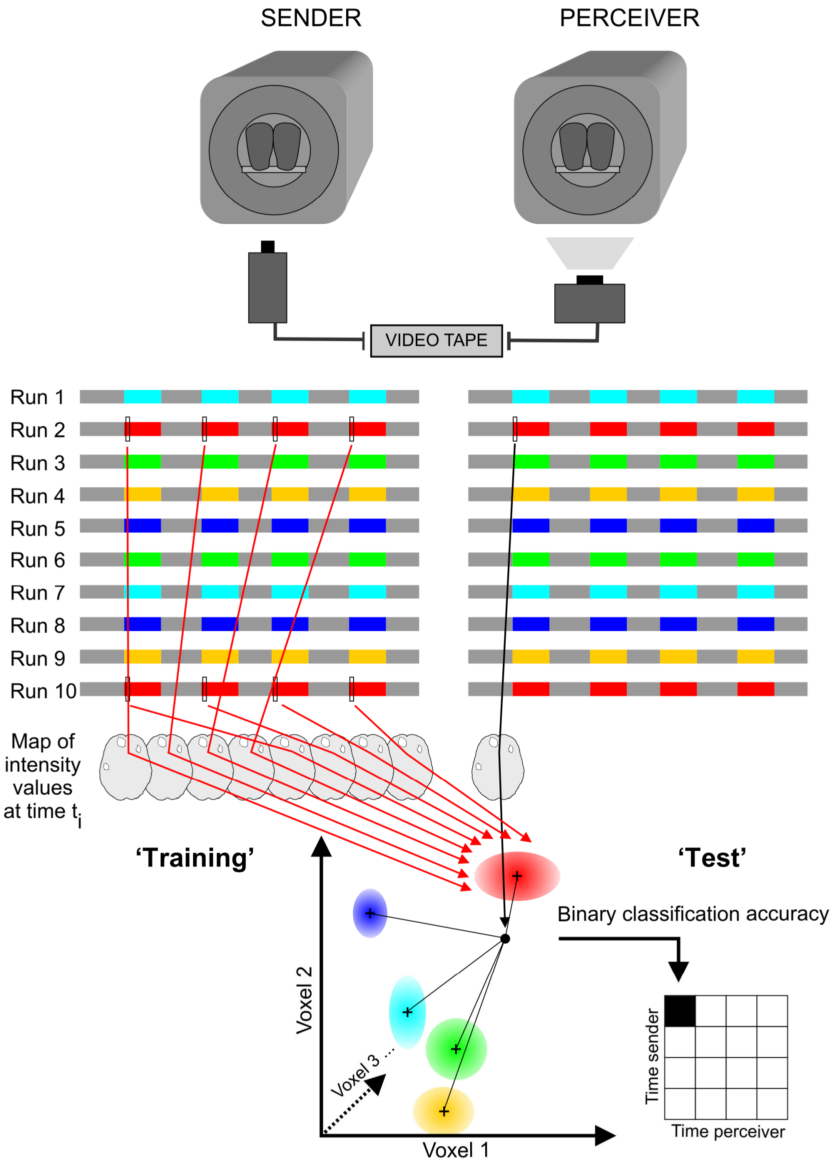

Figure S2. Time-resolved classification analysis. Classification was based on intensity values in single functional images. Information was combined across voxels that showed above-chance decoding accuracy in the voxel-wise analysis (i.e. the 'shared network of affect'). Please note that to avoid circularity, voxels within the shared network were identified separately for each sender-perceiver pair based on data from the remaining sender-perceiver pairs only. Intensity values of these voxels were represented as vectors in m-dimensional space, where m is the number of voxels. First, for each 2s-time window (corresponding to one functional scan), mean vectors were computed across the eight intensity vectors of each emotion (4 trials per run x 2 runs per emotion, indicated by the same colour) for the sender ('Training'). Second, these mean vectors were used to classify the perceiver's brain activity. The perceiver's response in a given 2s-time window was classified according to the smallest Euclidian distance in m-dimensional space (for visualization only the first two dimensions are shown) between the perceiver's response and these mean vectors ('Test'). Classification of the perceiver's brain activity was carried out separately for each emotion period and each combination of time windows, yielding a total of 40 binary classification accuracies (10 runs x 4 emotion periods per run) for each combination of time windows.

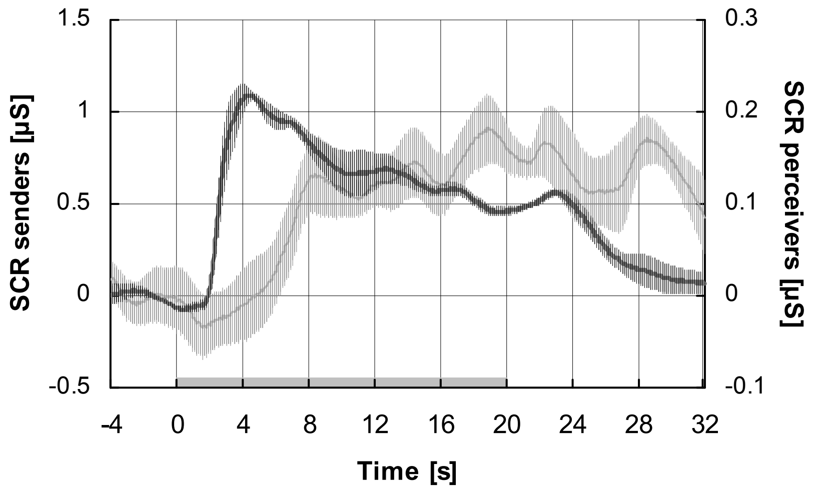

Figure S3. Skin conductance response (SCR) of senders and perceivers during facial communication of affect. Black, senders; grey perceivers. Bars represent the standard error of the mean at each sampling point. The light grey bar indicates the duration of the emotion period. Note the different scaling on the left and right y-axis.

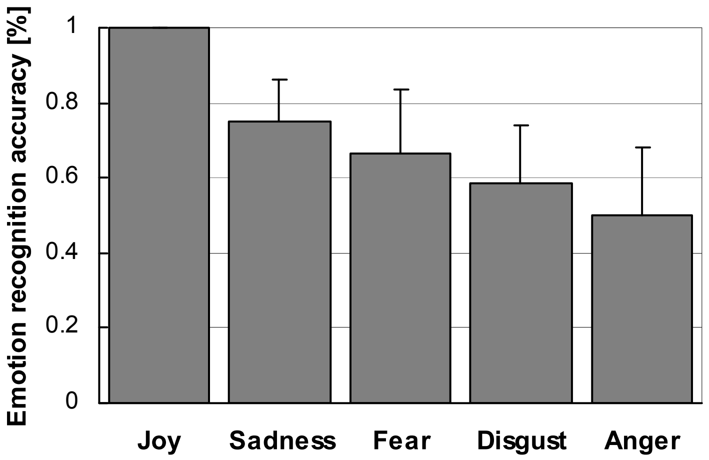

Figure S4. Verbal emotion recognition by perceivers. On average, perceivers recognized the sender's emotion in 70 percent of the runs. Recognition was above chance ($p = .20$) for every type of emotion (binomial $p < .05$) and there was no significant difference in recognition rates between emotion types (Chi-Square = 2.5, $df = 4$, $p > .50$).

Location of cluster	Coordinates at peak	T value at peak	Cluster size (k)	Voxels per anatomical structure	
**Temporo-insular cortex R**	33 6 -27	11.0	772	157	Superior temporal pole R
				94	Middle temporal pole R
				63	Parahippocampal gyrus R
				43	Rolandic operculum R
				39	Amygdala R
				38	Hippocampus R
				36	Superior temporal gyrus R
				26	Insula R
				25	Superior temporal gyrus R
				17	Fusiform gyrus R
				16	Inferior temporal gyrus R
				16	Middle temporal gyrus R
**Temporo-insular cortex L**	-33 6 -27	7.9	644	142	Superior temporal pole L
				54	Parahippocampal gyrus L
				52	Amygdala L
				47	Insula L
				37	Inferior frontal gyrus L
				36	Middle temporal gyrus L
				21	Middle temporal pole L
				15	Hippocampus L
				15	Superior temporal gyrus L
				14	Olfactory gyrus L
				12	Fusiform gyrus L
				12	Inferior temporal gyrus L
**Frontal cortex R**	30 24 48	6.4	242	169	Middle frontal gyrus R *
				46	Superior frontal gyrus R
**Medial parietal cortex L**	-15 -48 39	14.4	188	36	Middle cingulate cortex L
				31	Precuneus L *
				28	Posterior cingulate cortex L
**Parietal cortex L**	-63 -21 27	8.3	181	146	Postcentral gyrus L*
				19	Supramarginal gyrus L
**Temporal cortex R**	27 -63 -15	13.3	103	40	Fusiform gyrus R *
				25	Lingual Gyrus R
**Temporal cortex R**	60 -69 42	10.4	103	27	Angular gyrus R

Supplemental Table. Clusters in which the level of the perceiver's emotion-specific brain activity during affective communication could successfully be predicted from the level of the sender's brain activity (height threshold T = 3.3, p = .01, corrected at cluster level). Ordering is by size. Only anatomical structures that have at least 10 suprathreshold voxels are listed. Coordinates are in MNI space. Anatomical structures are labeled with the AAL atlas (Tzourio-Mazoyer et al., 2002). The anatomical structure that contains the highest activated voxel is marked with an asterisk. L, left hemisphere, R right hemisphere.

Instruction given to the sender just before scanning

[Original German wording]

‚Wir sind in dieser Studie ganz besonders an emotionaler Kommunikation interessiert. Wir nehmen an, dass emotionale Kommunikation die Gehirnaktivität in den Emotionsregionen des Gehirns zwischen Sender und Beobachter synchronisiert, dass also die Gehirnaktivität des Beobachters die des Senders widerspiegelt. Nach dieser Synchronizität suchen wir. Ihre Aufgabe ist es, sich in eine emotionale Situation hineinzuversetzen und Ihrem Partner zu zeigen, was Sie dabei empfinden. Natürlich können wir synchrone Gehirnaktivität in den Emotionsregionen nur dann beobachten, wenn der Sender und der Beobachter tatsächlich das gleiche empfinden. Es nützt also nichts, wenn Sie versuchen, Ihrem Partner eine Emotion vorzuspielen. Sie sollen versuchen, sich in eine emotionale Situation hineinzuversetzen und ein Gefühl nur dann zeigen, wenn Sie es wirklich empfinden.

Die Untersuchung besteht aus 10 Durchgängen, in denen Sie sich jeweils viermal für 20s in eine emotionale Situation versetzen sollen. Während dieser 20s sehen Sie auf einem Bildschirm ein entsprechendes Wort, zum Beispiel ‚Freude'. Sie müssen während dieser 20s nicht die ganze Zeit an dieselbe Situation denken, sondern können ruhig zwischen verschiedenen Situationen wechseln. Zwischen den emotionalen Phasen sollen Sie entspannen. Wenn Sie keine weiteren Fragen haben, würde ich jetzt mit Ihnen die verschiedenen Emotionen besprechen und Vorschläge machen, wie Sie sich vielleicht in diese Emotionen versetzen können.'

[English translation]

'In this study, we are especially interested in emotional communication. We think that emotional communication synchronizes brain activity in the emotional centres of the brain between sender and observer, i.e. that the brain activity of the observer reflects that of the sender. We are trying to find evidence for this synchronization. Your task is to put yourself in a certain emotional state and to show to your partner which emotions you feel whilst in this state. Of course we can observe synchronized brain activity in the emotional regions only if the sender and observer actually feel the same emotions. This means that trying to act out the emotion to your partner is no use. You should try to immerse yourself in a certain emotional state and exhibit a feeling only if you really feel it.

The experiment consists of 10 runs. In each run you should put yourself in a certain emotion situation four times, each lasting for 20s. During these 20s you will see an appropriate word on the screen, for example 'joy'. During these 20s, you don't have to think of the same situation for the whole time; you can switch between different situations. You should relax between these emotional phases. Unless you have further questions, I will now discuss the different emotions with you and make suggestions as to how you might put yourself in these emotional states.'

Instruction given to the perceiver just before scanning

[Original German wording]

‚Wir sind in dieser Studie ganz besonders an der Beobachtung von Mimik interessiert. Wir nehmen an, dass das Beobachten der Mimik die Gehirnaktivität zwischen Sender und Beobachter synchronisieren kann, dass also die Gehirnaktivität des Beobachters die des Senders widerspiegelt. Nach dieser Synchronizität suchen wir. Ihre Partnerin wird während der Untersuchung verschiedene Dinge erleben. Dabei gibt es auch Ruhephasen, wundern Sie sich also nicht, wenn manchmal eine Zeit lang nichts passiert. Ihre Aufgabe ist es, ihre Partnerin zu beobachten, und zu versuchen, mit ihrer Partnerin mitzuempfinden'.

[English translation]

'In this study, we are especially interested in the observation of facial expressions. We think that observation of facial expressions synchronizes brain activity between sender and observer, *i.e.* that the brain activity of the observer reflects that of the sender. We are trying to find evidence for this synchronization. Your partner will experience different things during scanning. There will also be some resting phases, so sometimes nothing will happen. Your task is to observe your partner and to try to feel with her'.
